# Velopharyngeal closure analysis using four-dimensional computed tomography: a pilot study of healthy volunteers and adult patients with cleft palate

**DOI:** 10.1186/s12880-019-0350-4

**Published:** 2019-07-08

**Authors:** Yoshikazu Kobayashi, Daisuke Kanamori, Naoko Fujii, Yumi Kataoka, Emiko Hirai, Satoshi Yoshioka, Koji Satoh, Hiroshi Toyama, Kensei Naito, Koichiro Matsuo

**Affiliations:** 1 Department of Dentistry and Oral-Maxillofacial Surgery, Fujita Health University, School of Medicine, 1-98, Dengakugakubo, Kutsukake, Toyoake, Aichi 470-1192 Japan; 2 Department of Dentistry, Nanakuri Memorial Hospital, Fujita Health University, 424-1, Oodoricho, Tsu, Mie 514-1295 Japan; 3 Department of Radiology, Bantane Hospital, Fujita Health University, 3-6-10, Otobashi, Nakagawa-ku, Nagoya, Aichi 454-8509 Japan; 4 Department of Radiology, Fujita Health University Hospital, 1-98, Dengakugakubo, Kutsukake, Toyoake, Aichi 470-1192 Japan; 5 Department of Otolaryngology, Fujita Health University, School of Medicine, 1-98, Dengakugakubo, Kutsukake, Toyoake, Aichi 470-1192 Japan; 6 Department of Radiology, Fujita Health University, School of Medicine, 1-98, Dengakugakubo, Kutsukake, Toyoake, Aichi 470-1192 Japan

**Keywords:** Velopharyngeal insufficiency, Nasopharyngoscopy, 320-row area detector computed tomography, Speech pathology, Palatoplasty, Kinematic analysis

## Abstract

**Background:**

Nasopharyngoscopy is a common method to evaluate velopharyngeal closure in patients with cleft palate. However, insertion of a fiberoptic nasopharyngoscope causes discomfort in patients. The aim of this study was to estimate the reliability of short-time exposure images obtained using 320-row area detector computed tomography (320-ADCT) as a novel evaluation method for the assessment of velopharyngeal function.

**Methods:**

We evaluated five healthy adult volunteers and five postoperative adult patients with cleft palate. During a 3.3-s imaging exposure, the participants were asked to perform two tasks: nasal inspiration and subsequent oral expiration through a catheter into a water-filled cup. The movement of the velopharyngeal structures was recorded during each examination, and the presence of velopharyngeal insufficiency (VPI) and velopharyngeal closure (VPC) patterns were estimated. If VPI was detected, the cross-sectional area was also calculated. Cohen’s kappa and weighted kappa coefficients were used to evaluate the concordance of nasopharyngoscopy and 320-ADCT evaluation.

**Results:**

Speech pathology evaluation did not reveal hypernasality in any study participant. Micro-VPI was detected by nasopharyngoscopy in one healthy volunteer and two patients. 320-ADCT detected micro-VPI in two more patients. The cross-sectional area of the VPI in these subjects ranged from 2.53 to 16.28 mm^2^. Nasopharyngoscopy and 320-ADCT were concordant in detecting VPI in eight participants (κ = 0.6) and in assessing VPC patterns in nine (κ = 0.82). Moreover, images obtained using 320-ADCT allowed for reduced dead angle and, thus, easy detection of micro-VPI and Passavant’s ridges.

**Conclusion:**

Although the radiation exposure cannot be ignored, our novel evaluation method using 320-ADCT enables more detailed evaluation of VPC than nasopharyngoscopy. Future studies should investigate the relationship between 320-ADCT findings and speech pathology evaluations.

**Electronic supplementary material:**

The online version of this article (10.1186/s12880-019-0350-4) contains supplementary material, which is available to authorized users.

## Background

Velopharyngeal insufficiency (VPI) is commonly observed in patients with cleft palate due to the structural anatomical abnormality of the soft palate. It can affect both speech and swallowing, which is of major concern in growing children [1]. Multiple surgical or nonsurgical approaches are used to improve velopharyngeal function (VPF), and various examinations are used to assess VPF. Although the evaluation of hypernasality by speech pathology is the gold standard for assessing VPF after cleft repair, an objective VPF analysis is essential for choosing the appropriate surgical procedure for secondary surgery for VPI. In addition to perceptual speech evaluation, the combination of lateral cephalogram and nasopharyngoscopy is commonly used for assessing the anatomical abnormalities causing VPI. However, lateral cephalogram only evaluates the two-dimensional (2D) elevation or thickness of the soft palate. Although nasopharyngoscopy is useful for assessing the anatomical abnormality, it can cause discomfort to the patient, due to the insertion of the fiberoptic nasopharyngoscope into the nasopharyngeal space, which increases the difficulty of the examination. Moreover, multiple examinations are time-consuming.

In 2008, the 320-row area detector computed tomography (320-ADCT) scanner first appeared as a medical imaging apparatus that could append the time phase to three-dimensional (3D) images, obtaining so-called “four-dimensional (4D) images [2, 3]”. This equipment loads a broad area detector (0.5 mm × 320 rows = 160 mm) and a cone-beam irradiation source onto a high-speed rotating gantry. It can thus provide wide-range 3D images without moving the patient’s bed and can generate 4D images of the same position continuously or intermittently. Therefore, it is of great significance in the evaluation of cardiovascular disease [3], dysphagia [4, 5], and cerebrovascular disease [6, 7].

In 2015, Sakamoto et al. first reported the clinical application of 320-ADCT to estimate VPF in patients with cleft palate [8]. The authors evaluated five children (aged 4–10 years) with persistent VPI post-palatoplasty with 10-s imaging, during vowel phonation and swallowing. They concluded that 320-ADCT provides detailed morphological and kinematic analysis of VPC and may offer advantages over conventional procedures [8]. However, the authors also advised that the high exposure dose during imaging (4.44 ± 1.64 mSv) could not be ignored in pediatric patients.

The aim of this pilot study was to estimate the reliability of short-time and dose-reduced exposure imaging by 320-ADCT as a novel evaluation method for the assessment of VPC before use in pediatric patients.

## Methods

### Subjects

We evaluated five healthy volunteers (two men and three women, age range 27 to 31 years) and five postoperative patients with cleft palate (four men and one woman, age range 20 to 24 years). All patients had undergone two-stage palatoplasty without pharyngeal flap operation.

The study was conducted in accordance with the Declaration of Helsinki (1964) and was approved by the Fujita Health University Ethics Review Committee (reference number: HM17–038). All participants were sufficiently informed about the purpose of this study, including the fact that it did not involve therapeutic, but exploratory research, and provided written informed consent. The participants were also informed of the estimated radiation exposure dose by 320-ADCT (about 1.65 mSv), as cited in earlier research [9].

### Procedure

Speech samples, including single sounds, syllable repetition, and connected speech, were corrected for speech pathology evaluation. Three well-trained speech-language-hearing therapists assessed the existence of hypernasality based on these samples.

320-ADCT (Aquilion ONE™/Genesis Edition. Toshiba Medical Systems Corp., Tochigi, Japan) and nasopharyngoscopy were also performed for all the participants. Participants were asked to perform two successive tasks: nasal inspiration and subsequent oral expiration through a catheter into a water-filled cup.

**Fig. 1:**
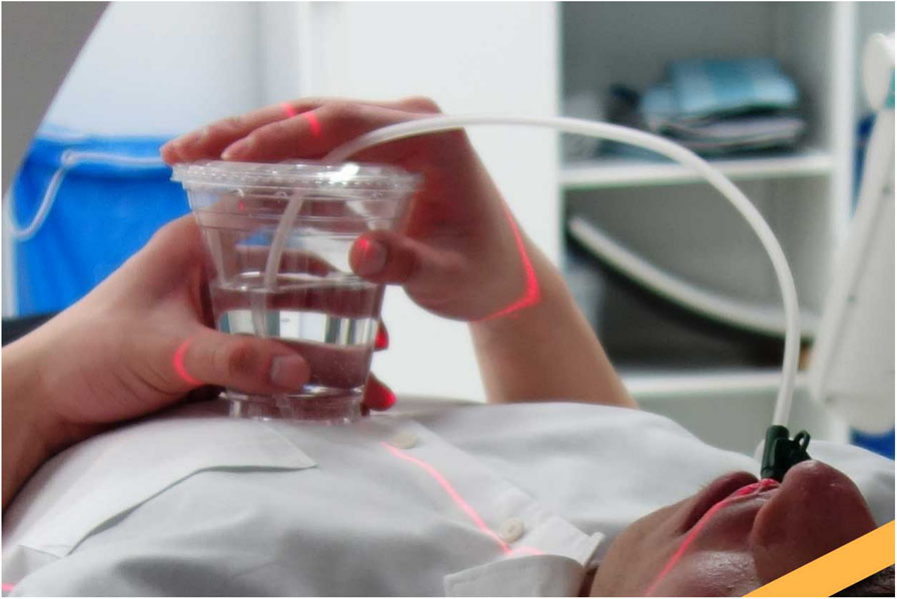
A participant performing the study’s tasks (Authors’ original picture). Each participant performed two tasks: nasal inspiration and subsequent forceful expiration through a catheter into a water-filled cup

During the CT scan, each subject lay down horizontally on the bed and performed the task, following a signal given by an examiner standing beside the CT scanner. Scanning was started immediately after the onset of inspiration. Expiration was continued 1 sec after the start and until the end of the scanning. We used a 14-French gauge (4.7-mm outer diameter, 47-cm long) catheter (Nurvie™, Covidien Japan, Tokyo) and disposable clear PET cups (370 mL) half-filled with water (Fig. 1). This ensured that the examiner could easily ascertain that the task was being performed, by the formation of bubbles in the water.

**Fig. 2:**
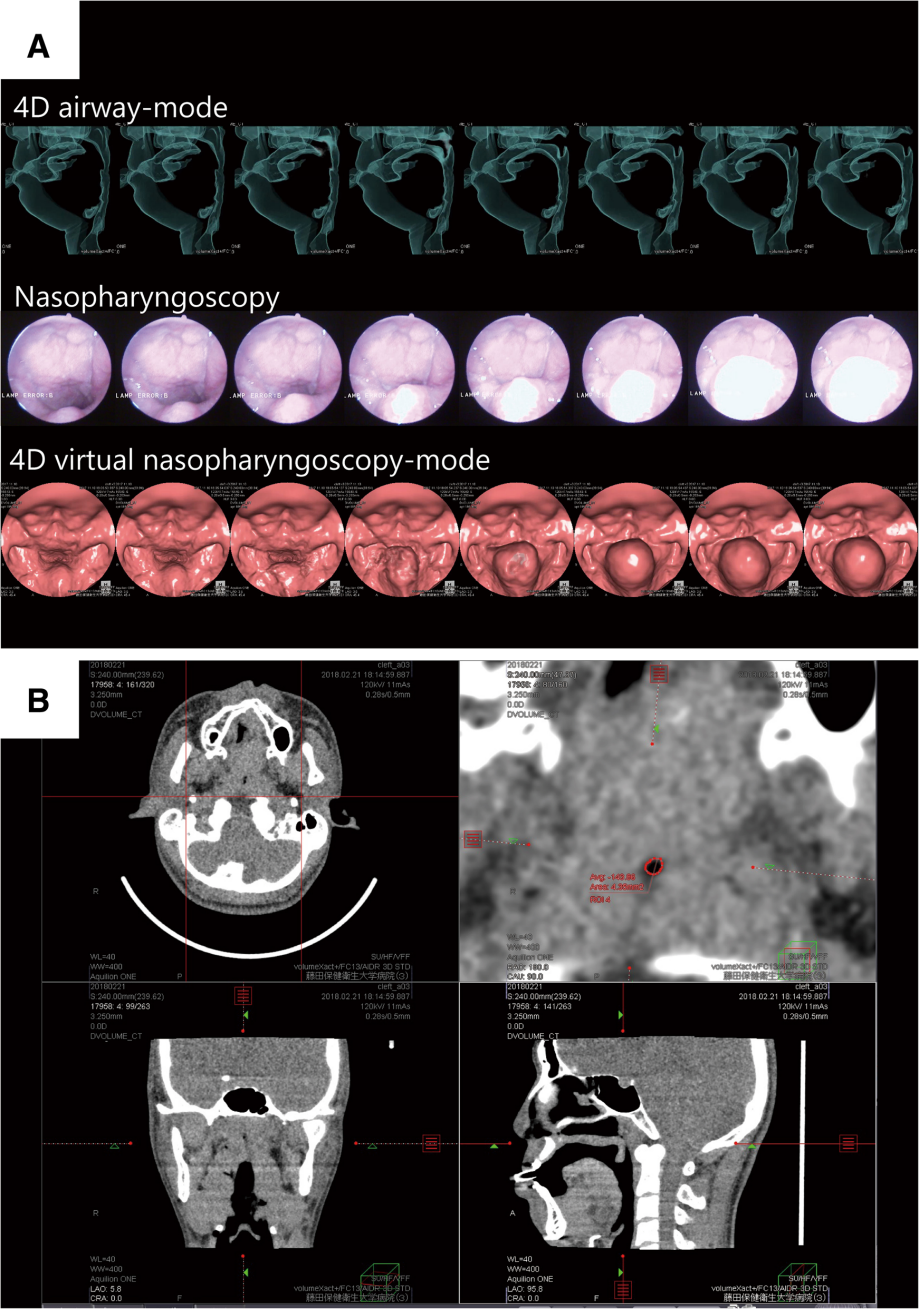
Computed tomography and nasopharyngoscopy images. **a** Images of the airway in 4D-mode (top), actual images of nasopharyngoscopy (middle), and 4D images of virtual nasopharyngoscopy-mode (bottom) **b** Multiplanar reconstruction images to calculate the cross-sectional area of velopharyngeal insufficiency

The scanning parameters were set as follows: field of view, 160 mm; tube voltage, 120 kV; tube current, 60 mA; and exposure time, 3.30 s (0.275 s/rotation × 12 rotations). To improve the temporal resolution, we implemented a half-reconstruction technique by which images were generated from 0.138-s data for image generation. The images were drawn with 0.5-mm slice thickness and transferred to a medical imaging workstation (Ziostation2, Ziosoft, Inc., Tokyo, Japan) where 4D images of airway mobility (airway-mode), 4D images in virtual endoscopy mode, and multiplanar reconstruction (MPR) images were generated (Fig. 2). Airway-mode, virtual endoscopy-mode, and MPR images had a window length of 500, 75, and 40 HU, and a window width of 500, 500, and 400 HU, respectively.

Nasopharyngoscopy was performed by two well-trained otorhinolaryngologists. During the examination, subjects lay down on the bed in the same position used during the CT procedure.

**Fig. 3:**
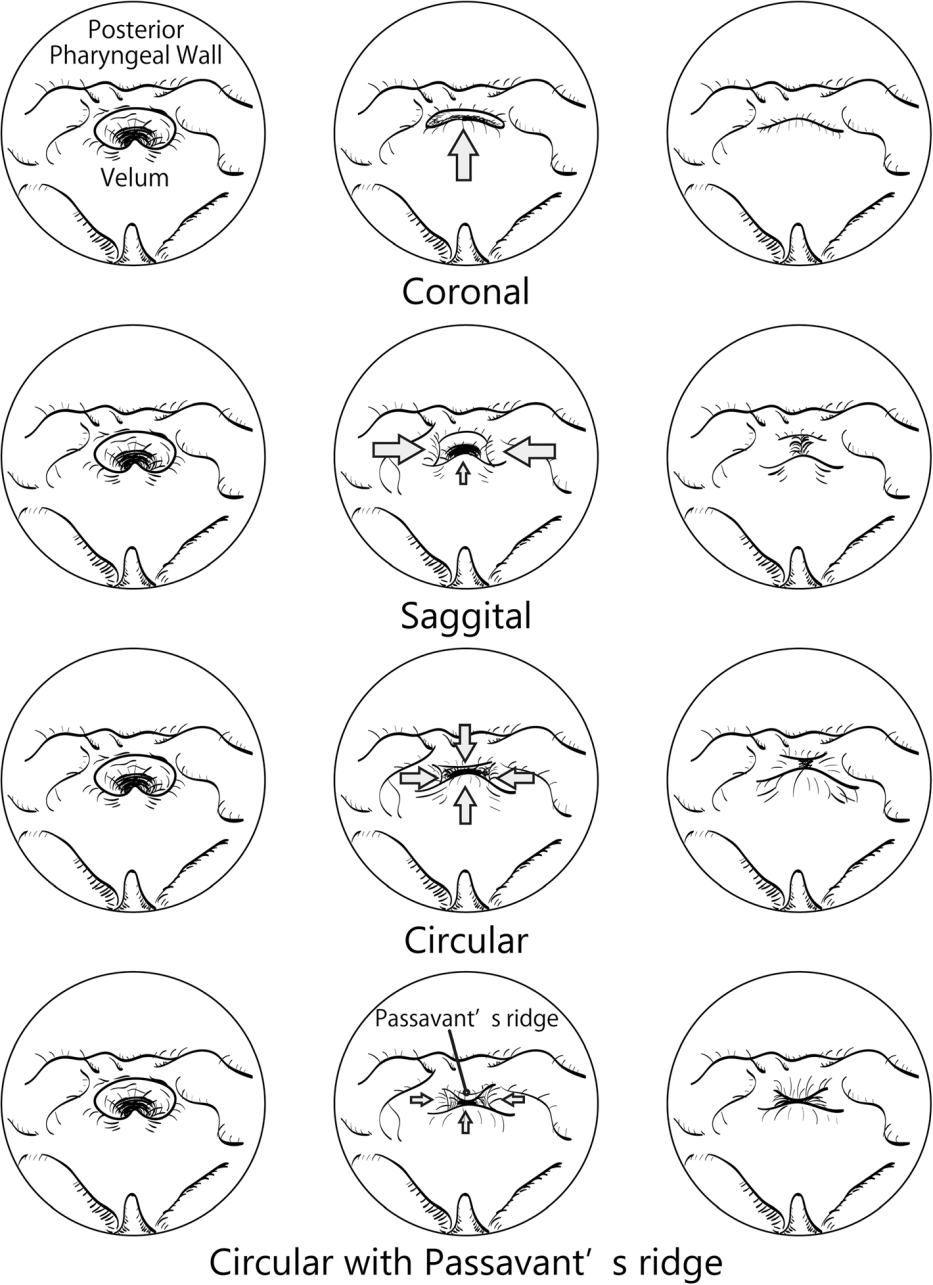
Patterns of velopharyngeal closure. The patterns of velopharyngeal closure are categorized into four groups in accordance with previous literature [10]


The movement of the velopharyngeal structures was recorded during each examination (Additional file 1: Video S1), and the presence of VPI and VPC patterns were estimated. VPC patterns were categorized into four groups: coronal, sagittal, circular, and circular with Passavant’s ridge, in accordance with earlier reports [10] (Fig. 3). The minimum cross-sectional area of the nasopharynx was measured parallel to the palatal plane, using MPR images obtained during blowing. The volume of the CT dose index (CTDI_vol_) and dose length product (DLP) for each scan were around 16.10 mGy and 258.80 mGy·cm, respectively.

**Additional file 1:** **Video S1.** The actual footage of 4D-CT obtained from volunteer #3. (WMV 2806 kb)


### Statistical analysis

We assessed the consistency of the results obtained by CT and nasopharyngoscopy in detecting the presence of VPI, as the primary outcome measure, using Cohen’s kappa coefficient. Moreover, the consistency of the two methods for detecting VPC patterns was evaluated using the weighted kappa coefficient. We also evaluated consistency of these two methods, with respect to VPI and hypernasality. Moreover, we assessed the timing of the movements of the soft palate, lateral pharyngeal wall (LPW), posterior pharyngeal wall (PPW), and tongue, to evaluate the motor coordination of the anatomical structures associated with the VPC.

Statistical analysis was conducted using JMP® v. 13.2 (SAS Institute Inc., Cary, NC, USA), and statistical significance was accepted when *p* < 0.05.

## Results

Micro-VPI was detected by nasopharyngoscopy in one healthy volunteer and in two patients with cleft palate (Table 1).

**Table 1:** Distribution of velopharyngeal insufficiency and patterns of velopharyngeal closure

			Nasopharyngoscopy		CT evaluation		
No.	age	sex	VPI	VPC pattern	VPI	VPC pattern	VPI cross-sectional area (mm^2^)
Volunteer #1	31	M	+	circular	+	circular	3.09
Volunteer #2	31	F	–	circular	–	circular	0
Volunteer #3	29	M	–	coronal	–	coronal	0
Volunteer #4	27	F	–	circular	–	circular	0
Volunteer #5	30	F	–	circular	–	circular	0
Adult patient #1	24	M	–	circular	+	circular with Passavant’s ridge	6.22
Adult patient #2	21	M	+	circular	+	circular	16.28
Adult patient #3	22	M	+	circular with Passavant’s ridge	+	circular with Passavant’s ridge	4.35
Adult patient #4	20	M	–	circular with Passavant’s ridge	–	circular with Passavant’s ridge	0
Adult patient #5	21	F	–	circular with Passavant’s ridge	+	circular with Passavant’s ridge	2.53

*CT* computed tomography, *VPI* velopharyngeal insufficiency, *VPC* velopharyngeal closure; +, present; −, absent

**Fig. 4:**
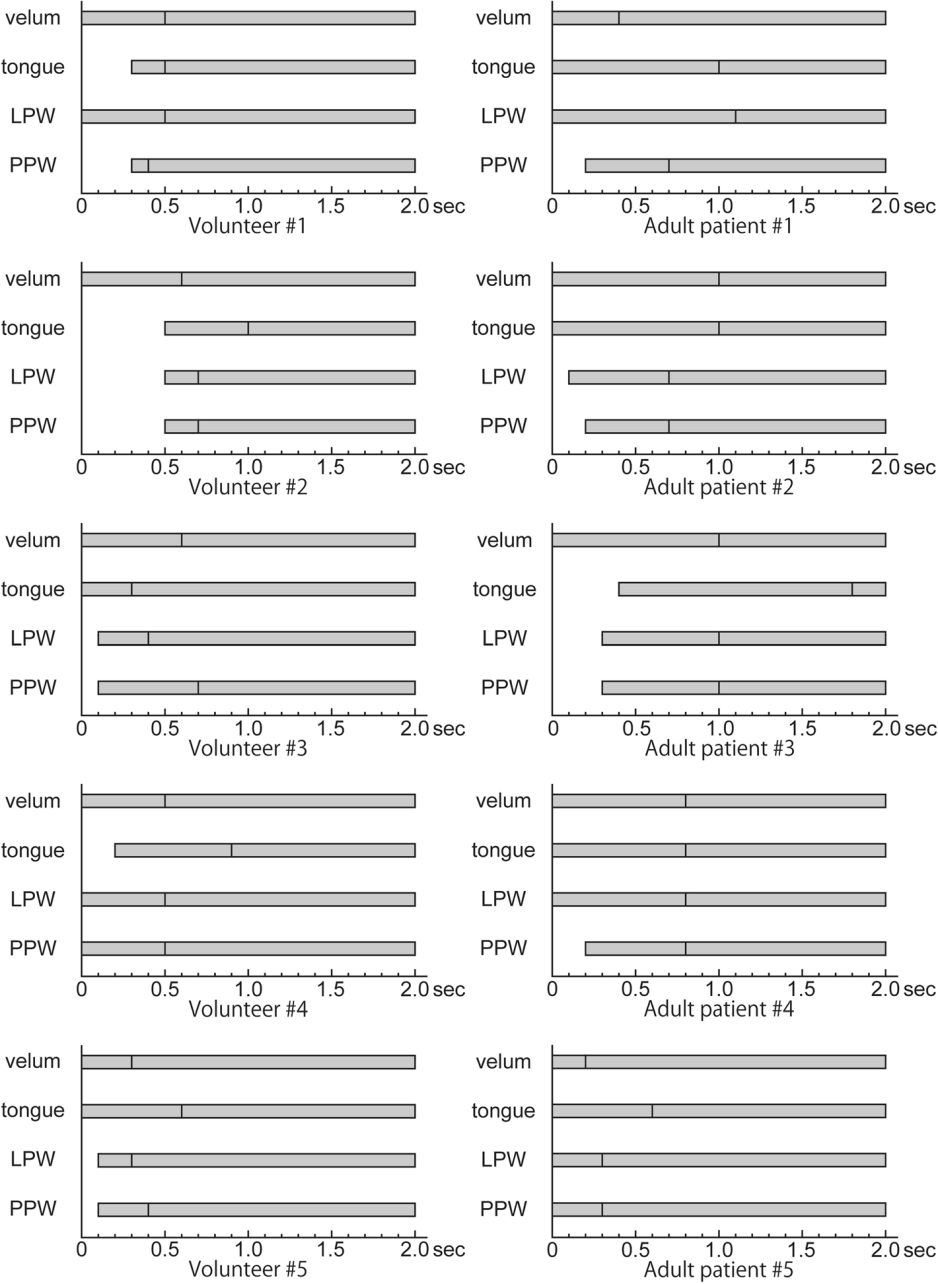
Motor coordination of anatomical structures associated with velopharyngeal closure in each participant. Each crossbar indicates the continuity of motion. The vertical line in each crossbar indicates the onset of motion. LPW, lateral pharyngeal wall; PPW, post pharyngeal wall

320-ADCT detected micro-VPI in two more patients. The cross-sectional area of VPI in these five participants ranged from 2.53–16.28 mm^2^. Nasopharyngoscopy and 320-ADCT were concordant in their assessment in 8/10 participants (κ = 0.6, *p* = 0.04), and in assessing the VPC patterns in 9/10 participants (κ = 0.82, *p* = 0.0009). Hypernasality was not observed in any of the cases, even though, some subjects exhibited the presence of VPI. Passavant’s ridges were seen in four postoperative patients. The motor coordination of the anatomical structures associated with VPC for each subject is indicated in Fig. 4.

## Discussion

In this study, we estimated the reliability of a novel evaluation method, “virtual nasopharyngoscopy,” for the assessment of VPC, using short-time exposure 320-ADCT.

Although nasopharyngoscopy provides useful 3D information, allowing for the detection of the region and the severity of VPI, it causes pain and distress to younger children or patients, as it induces a vomiting reflex. Thus, in such cases, surgical intervention for VPI, performed without presurgical nasopharyngoscopy, must rely on the empirical assumptions of skilled surgeons. Moreover, incomplete visualization due to the dead angle created by the elevated soft palate is an obstacle during evaluation [11]. Several studies have estimated VPI or VPF using dynamic magnetic resonance imaging. Although it is a non-invasive technique, which can be repeated without the risk of radiation exposure, its low imaging speed, narrow spatial coverage, and noise may interfere in the examination of pediatric patients, when compared to CT [12, 13].

The first assessment of VPF with 320-ADCT in patients with cleft palate was reported in 2015 by Sakamoto et al. [8]. In that study, the authors evaluated five children with persistent VPI, following palatoplasty, using three tasks during a ~ 10-s CT exposure: sustained production of the vowels [/a:/] and [/i:/], and swallowing. They concluded that 4D-CT produces clear and detailed images with lesser stress and pain, shorter examination duration than other imaging modalities, and quantitative VPC evaluation [8]. However, they reported that the radiation exposure of approximately 4 mSv, which is larger than that required in cephalometry, videofluoroscopy, and conventional CT [8], was a major drawback of this procedure. In our study, we modified the 320-ADCT scan protocol to shorten the exposure time during VPF assessment. Thus, the existence of VPI and VPC patterns was precisely assessed, consistent with the findings of nasopharyngoscopy. Furthermore, we implemented a quantitative calculation of the nasopharyngeal cross-sectional area and dynamically analyzed the anatomical structures associated with VPC, which was not possible earlier, with the sole use of an existing modality. Although none of the subjects in our study exhibited hypernasality during speech evaluation, we detected micro VPI in five out of ten participants, which was not detected by nasopharyngoscopy in two of these participants.

The International Commission on Radiological Protection and several other international organizations have cooperated to find ways to minimize radiation exposure. Diagnostic reference levels are important methods for optimizing protection, including the computed tomography dose index (CTDI_vol_) and dose length product (DLP). The CTDI_vol_ is a dose index that evaluates the performance of CT scanners, while the DLP is the product of CTDI_vol_ and scan length that estimates the total amount of radiation [14]. In 2015, the Japan Network for Research and Information on Medical Exposure published the report “Diagnostic Reference Levels Based on Latest Surveys in Japan.” [15] In this report, the CTDI_vol_ for head and chest CT for children aged 6–10 years was set at 60 and 15 mGy, respectively, while the corresponding DLP was set at 850 and 410 mGy·cm. In our study, the values of CTDI_vol_ and DLP were 16.10 mGy and 258.80 mGy·cm, respectively. Thus, reducing the exposure dose is of the outmost importance when this method is used in pediatric patients.

In this study, all the participants could perform the requisite tasks during the CT examination after a few rehearsals, except for the first participant, a healthy volunteer, who missed the examiner’s sign. This dynamic analysis allowed for some extent of timing errors. This is the most important advantage of this method, as it can be useful during the functional examination of pediatric patients, who have difficulties in performing tasks depending on the examiners’ signs.

Moreover, we demonstrated that 4D-CT provides kinematic analysis of the individual anatomical structures associated with VPC. To the best of our knowledge, there are no reports of this time-based analysis in the literature. Based on this analysis, we found that the velum starts to move before the other VPC structures, always, regardless of the presence of VPI or VPC patterns. Future research should address whether these kinematic features affect or cause hypernasality.

This study has some limitations. First, the number of feasible tasks that could be performed during this short-duration of CT scanning was very limited; therefore, some discrepancies might exist between the results obtained from this method and the actual hypernasality. In fact, in this study, we detected micro-VPI in five participants, although none of the participants exhibited hypernasality. The presence or absence of the influence of micro-VPI on hypernasality is interesting, which may be elucidated quantitatively in future studies. Second, the exposure dose used in this method remains higher than the dose used in conventional radiological assessments, such as cephalometry or videofluoroscopy. However, we believe that the exposure time or scanning range could be further improved. Nevertheless, the data obtained using this method are acceptable in selected situations, as in cases of children who cannot receive nasopharyngoscopy or to estimate the size of the flap in those who need to undergo pharyngeal flap surgery. Of course, the largest advantages of CT evaluation when compared to non-irradiating methods are reproducibility, standardization, and a quantitative analysis. We believe that further research will make the best use of these merits and improve the quality of treatment and management in cleft repair cases.

## Conclusion

Nasopharyngoscopy and 320-ADCT have a high concordance rate for evaluating VPI, as well as VPC patterns. Images obtained using 320-ADCT exhibit reduced dead angle, allowing for easy detection of micro-VPI and Passavant’s ridge. Although some extent of radiation exposure cannot be ignored, our novel evaluation method using 320-ADCT allows for a more detailed evaluation of VPC than nasopharyngoscopy. Future studies should investigate the relationship between findings from 320-ADCT images and those from speech pathology evaluations.

## Data Availability

The datasets supporting the conclusions of this article are available from the corresponding author on reasonable request.

## Abbreviations

2D: Two-dimensional
320-ADCT: 320-row area detector computed tomography
3D: Three-dimensional
4D: Four-dimensional
CTDI_vol_: Volume of the CT dose index
DLP: Dose length product
LPW: Lateral pharyngeal wall
MPR: Multiplanar reconstruction
PPW: Posterior pharyngeal wall
VPC: Velopharyngeal closure
VPF: Velopharyngeal function
VPI: Velopharyngeal insufficiency
